# Water–lipid interface in lipidic mesophases with excess water†

**DOI:** 10.1039/d3fd00118k

**Published:** 2023-06-29

**Authors:** Yang Yao, Sara Catalini, Paolo Foggi, Raffaele Mezzenga

**Affiliations:** a Department of Health Sciences and Technology, ETH Zürich 8092 Zürich Switzerland raffaele.mezzenga@hest.ethz.ch; b European Laboratory for Non-Linear Spectroscopy, LENS 50019 Florence Italy; c Department of Physic and Geology, University of Perugia 06123 Perugia Italy; d CNR-INO, National Research Council-National Institute of Optics 50125 Florence Italy; e Department of Chemistry, Biology and Biotechnology, University of Perugia 06123 Perugia Italy; f Department of Materials, ETH Zürich 8093 Zürich Switzerland

## Abstract

This study investigates the influence of excess water on the lipidic mesophase during the phase transition from diamond cubic phase (*Pn*3̄*m*) to reverse hexagonal phase (*H*_II_). Using a combination of small angle X-ray scattering (SAXS), broadband dielectric spectroscopy (BDS), and Fourier transform infrared (FTIR) techniques, we explore the dynamics of lipids and their interaction with water during phase transition. Our BDS results reveal three relaxation processes originating from lipids, all of which exhibit a kink during the phase transition. With the excess water, these processes accelerate due to the plasticizing effect of water. Additionally, our results demonstrate that the headgroups in the *H*_II_ phase are more densely packed than those in the *Pn*3̄*m* phase, which agrees with the FTIR results. Meanwhile, we investigate the influence of excess water on the lipid headgroups, the H-bond network of water, the lipid tail, and the interface carbonyl group between the head and tail of the lipid molecule. The results indicate that excess water permeates the lipid interface and forms additional hydrogen bonds with the carbonyl groups. As a result, the headgroups are more flexible in a lipidic mesophase with excess water than those in mesophases without excess water.

## Abstract 2

We study the influence of excess water on the phase transition of lipidic mesophases from the cubic to reverse hexagonal phase. Excess water permeates the interface and forms additional hydrogen bonds with lipids, accelerating the lipid dynamics.

## Graphical abstract

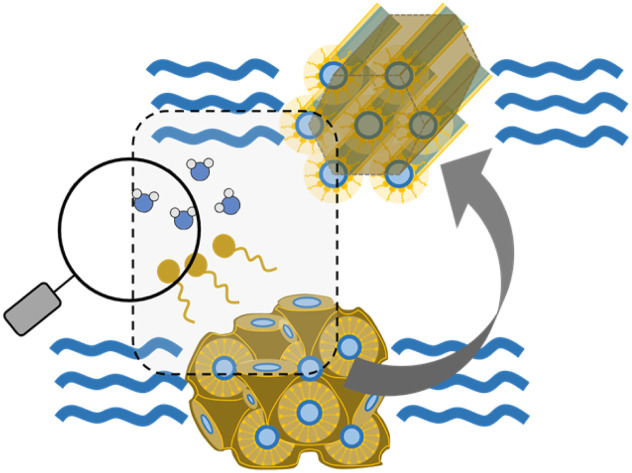

## Introduction

Lipidic mesophases, also known as lyotropic liquid crystals, have attracted considerable attention in both fundamental research and industrial applications due to their unique structural features and amphiphilic property.^1–3^ Typically, these mesophases are formed from the self-assembly of fatty acids (lipids) in water. Depending on the water content, amphiphilicity of lipids, and specific external conditions (*e.g.*, temperature), a wide range of phase structures can be formed, including lamellar phase (*L*_*α*_), cubic phases (gyroid, *Ia*3̄d; diamond, *Pn*3̄*m*; primitive, *Im*3̄*m*), reverse hexagonal (*H*_II_), and reverse micelle (*L*_2_).^4–6^ In all these phases, water is confined within nano-sized geometries while surrounded by lipids. The unique structural organization of confined water and lipids within these mesophases results in extended interfaces between hydrophilic and hydrophobic regions. At the same time, the large interfacial area in lipidic mesophases facilitates interactions between molecules, such as solutes,^7^ enzymes,^8,9^ and other amphiphilic species,^10^ leading to potential applications in drug delivery,^11,12^ cosmetics, and the food industry. In this context, understanding the physical properties of confined water and molecular interactions within the lipidic mesophase is essential.

Water under nano-sized confinement exhibits distinct behaviours compared to bulk water^13–16^ with respect to the hydrogen-bond (H-bond) networks, molecular dynamics, diffusion, and crystallization, owing to both the geometric constraints and its interaction with interfaces. An intriguing example of the unique properties of confined water within the lipidic mesophase is the detection of liquid water at the remarkably low temperature of −120 °C.^17,18^ In addition, researchers have been using Fourier transform infrared (FTIR) and dielectric radiation spectroscopy (DRS) to study the H-bond networks and molecular dynamics of water confined in diverse mesophases, as well as during the phase transition.^19–22^ Extensive studies have demonstrated two dynamically different fractions of water in the lipidic mesophase: interfacial bound water and interstitial bulk-like water. The interfacial bound water exhibits significantly slower dynamics compared to the interstitial water that is located further away from the interface.^19–21^ In our recent studies, we have employed broadband dielectric spectroscopy (BDS) to investigate the molecular dynamics of lipids within different phases and studied their interaction with confined water. One notable finding was the plasticizer effect of water on the lipidic mesophase observed from the faster lipid dynamics and decreased glass transition temperature of lipids.^23^

In lipidic mesophases with relatively low water content, the amphiphilic lipids can effectively encapsulate water within the mesophase structures. However, as the water content increases, a saturation point is reached, limiting the further entry of water into the mesophase. Beyond this critical threshold, any additional water will accumulate outside the mesophases as excess water.^24^ The understanding of the physical behavior of lipidic mesophases in the presence of excess water and their interactions is crucial for the application of bulk lipidic mesophases as well as for the design of lipidic mesophase nanoparticles such as cubosomes and hexosomes.^25–28^ In these systems, the lipidic mesophases are dispersed in aqueous solutions and stabilized by additional surfactants. Despite its significance, the behavior of the lipidic mesophase coexisting with excess water has not been systematically studied.

Herein, we investigate the dynamics of lipidic mesophases in the presence of excess water and study their interactions. To achieve this, small angle X-ray scattering (SAXS), BDS and FTIR techniques are employed. By comparing the lipidic mesophase with and without excess water, we observe the effect of excess water on the molecular dynamics of lipids during the phase transition from the diamond cubic phase (*Pn*3̄*m*) to reverse hexagonal phase (*H*_II_). The BDS findings revealed the presence of three distinct relaxation processes originating from the lipids in both samples with and without excess water. Interestingly, these relaxations exhibit a noticeable kink during the phase transition, suggesting a denser packing of lipid headgroups in the *H*_II_ phase than that in the *Pn*3̄*m* phase. In addition, in the presence of excess water, all these relaxations are accelerated, indicating a plasticizer effect of water on the lipidic mesophase. Furthermore, the combination of BDS and FTIR provides deep insights into the interaction between the lipidic mesophase and excess water. We examined the impact of excess water on the headgroups and tails of the lipids, as well as the H-bond network of water in the system. In addition, we conduct detailed analysis of the carbonyl group at the interface between the headgroup and tail of the lipid molecule.

## Results and discussion

### Phase structure and phase diagram

**Fig. 1:**
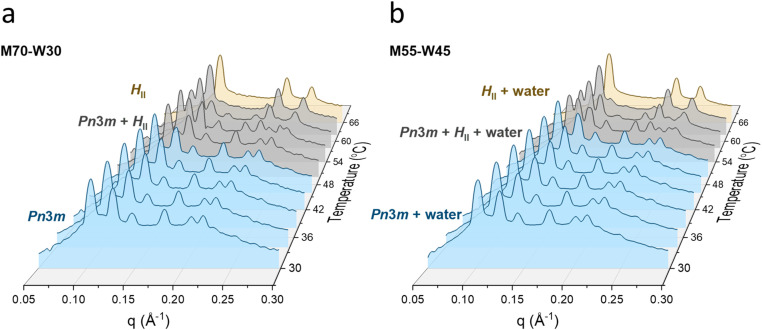
SAXS profiles of (a) M70-W30 and (b) M55-W45 as a function of temperature.

The phase structures of the monolinolein–water mesophase are studied by SAXS in the temperature range 30 to 66 °C. Fig. 1 shows the temperature dependence of the SAXS profile of samples M70-W30 and M55-W45. At 30 °C, the profile of both samples present Bragg reflections with relative positions with ratios of √2 : √3 : √4 : √6 : √8 : √9, reflecting a *Pn*3̄*m* cubic phase. While at 66 °C, the Bragg reflections in the spectra of both samples have ratios of √1 : √3 : √4, which reflects a reverse hexagonal phase (*H*_II_). From 50 to 62 °C, the phase transition from the *Pn*3̄*m* cubic to *H*_II_ phase occurs in both samples. The peak positions of samples with lower water fraction shift to lower *q* vectors as the water content increases at a fixed temperature (Fig. S1†). This suggests that water enters the mesophase and increases the lattice parameters. However, as the water content reaches a point, the lattice parameter of the mesophase remains unchanged upon further addition of water, indicating that excess water can no longer enter the mesophase and instead coexists with it (Fig. S1†). In the present monolinolein–water system with 45 wt% water, the lipidic mesophase is in equilibrium with excess water in the temperature range 30 to 66 °C.

**Fig. 2:**
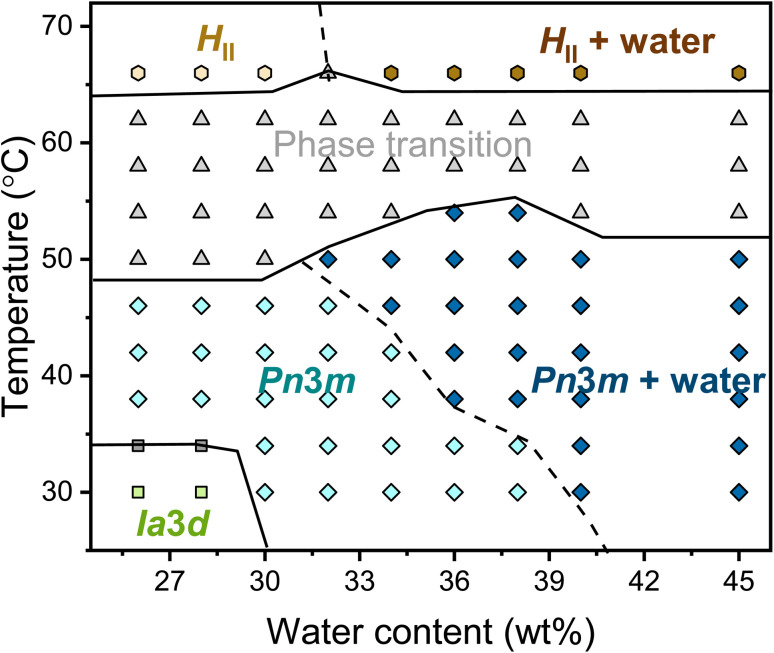
Phase diagram of the monolinolein–water mesophase as a function of water content and temperature obtained by SAXS. Phase labels: *Ia*3̄*d*, green squares; *Ia*3̄*d* + *Pn*3̄*m*, grey squares; *Pn*3̄*m*, light blue diamond; *Pn*3̄*m* + water, dark blue diamond; phase transition, grey up-pointing triangles; *H*_II_, light yellow hexagons; *H*_II_ + water, dark yellow hexagons. The lines dividing the phase regions are a guide to the eye. A dashed line separates the mesophases with and without excess water.

The complete temperature-content phase diagram of the binary monolinolein–water system was established by SAXS (Fig. 2). The previously published monolinolein–water phase diagram is available in the literature.^29^ However, it should be noted that the commercial dimodan (monolinolein) can vary from batch to batch and that its purity is that of an industrial grade product. Therefore, in our study, we carefully prepared samples with precise water content differences. Subsequently, we performed SAXS measurements on our specific batch of dimodan samples and used these results to plot an updated phase diagram. (Fig. S2†) typically, the phase transition from a cubic phase to a reverse hexagonal phase occurs as the temperature increases, passing through a temperature range where both phases coexist. At lower temperatures (<34 °C) and with lower water content (<30 wt%) in the sample, the system presents in the *Ia*3̄*d* cubic phase. While with higher water content (>30 wt%) at higher temperatures (30 < *T* < 50 °C), the system presents in the *Pn*3̄*m* cubic phase. At high water content (≥40 wt% at 30 °C), the *Pn*3̄*m* cubic phase coexists with excess water. It is worth noting that the boundary between *Pn*3̄*m* and *Pn*3̄*m* + water shifts to lower hydration as the temperature increases, meaning that the maximum possible water content inside the mesophase decreases with increasing temperature. The indications of phase boundaries are reasonably accurate except for the *Pn*3̄*m*/*H*_II_ coexisting region because of the mixed reflections contributing from both phases. Therefore, the boundary between *Pn*3̄*m* + *H*_II_ and *Pn*3̄*m* + *H*_II_ + water is plotted with the dashed line. At a higher temperature (∼66 °C), the transition from *Pn*3̄*m* cubic phase to *H*_II_ phase is completed with three identified reflections with ratio √1 : √3 : √4.

### Molecular dynamics of lipids during phase transition

**Fig. 3:**
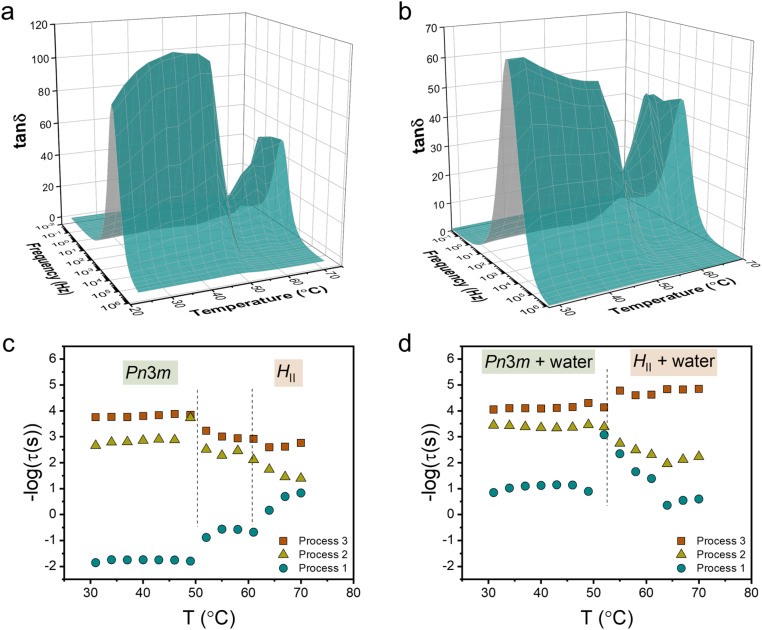
3D dielectric spectra of tan *δ* as a function of frequency and temperature for (a) M70-W30 and (b) M55-W45 lipidic mesophases. Relaxation times obtained from fitting tan *δ* to the (c) M70-W30 and (d) M55-W45 lipidic mesophase. The dashed lines indicate the phase transition temperatures obtained from SAXS.

We studied the molecular dynamics of lipids during the phase transition using broadband dielectric spectroscopy (BDS). The BDS spectra of M70-W30 and M55-W45 in the representation of tan *δ* (tan *δ* = *ε*′′/*ε*′), as a function of frequency in the temperature range 30–70 °C are shown in Fig. 3a and b, respectively. The phase transition is clearly observed from the significant fluctuation in the 3D plots of both samples, occurring in a similar temperature range as detected by SAXS. The relaxation times of individual processes at different temperatures are obtained from fitting the spectra with a combination of several Havriliak–Negami (H–N) models. In our previous study,^23^ we discussed the findings obtained from the analysis of the BDS spectra of the M70-W30 sample (without excess water). The results revealed the presence of three relaxation processes originating from lipids with distinct temperature dependencies. Notably, all three processes show a kink during the phase transition from the *Pn*3̄*m* cubic phase to the *H*_II_ phase. The slowest process (process 1) was found to be associated with the rheology of the system. On the other hand, the faster two processes, process 3 and process 2, were attributed to the headgroup association/reorientation and the interaction of polar headgroups within the matrix of the lipidic mesophase, respectively.

One aim of our current study is to investigate the effect of excess water on the molecular dynamics of the lipids during the phase transition. In line with our findings in M70-W30, we detected the presence of three processes in M55-W45 (Fig. 3c and d). However, all the processes become faster in M55-W45 compared to M70-W30 resulting from the plasticizer effect of water accelerating the overall dynamics in the system. The impact of excess water on the molecular dynamics of the lipids is particularly pronounced in process 1. In M70-W30, the relaxation time for this process ranges from 0.1 to 100 seconds. However, in M55-W45, the relaxation time significantly decrease to a range of 0.001 to 0.1 seconds. This substantial reduction in relaxation time highlights the enhanced dynamics resulting from the plasticizing effect of water in M55-W45 compared to M70-W30.

In M55-W45, we also observed that process 2 becomes slower in the *H*_II_ phase compared to the *Pn*3̄*m* cubic phase, similar to the observation in M70-W30. This change in dynamics can be attributed to the denser packing of the headgroups in the *H*_II_ phase, which leads to a slower relaxation process. Contrary to the findings in M70-W30, the fastest process, process 3, in M55-W45 becomes slightly faster in the *H*_II_ phase compared to that in the *Pn*3̄*m* cubic phase. This behaviour can be attributed to the substantially enhanced matrix dynamics in the presence of excess water.

### Vibrational assignment of molecular groups of the monolinolein–water mesophase

**Fig. 4:**
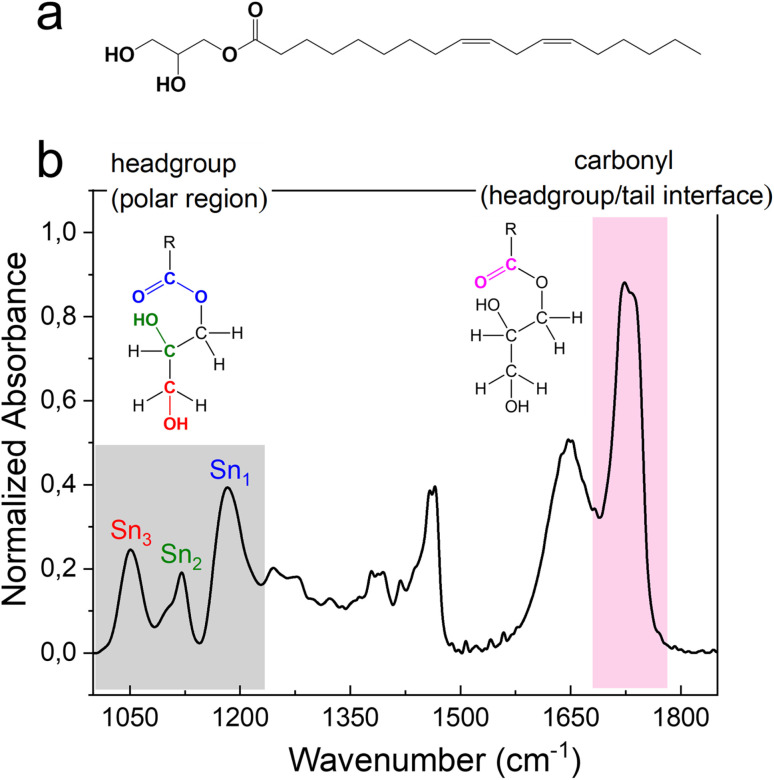
(a) Molecular structure of monolinolein. (b) FTIR spectra in the region 1000–1850 cm^−1^ of M70-W30 obtained at 30 °C. The Sn_1_, Sn_2_, Sn_3_ in the grey area indicate the corresponding vibrational motions associated to the headgroup. The headgroup/tail interface, ester C=O stretching at ∼1730 cm^−1^, is highlighted in pink.

The FTIR signals of headgroups and carbonyl interface fall in the wavenumber range 1000–1230 cm^−1^ and 1680–1780 cm^−1^, respectively (Fig. 4). The two signals deriving from the C–OH stretching vibrational motion on the polar head of monolinolein are indicated in Fig. 4b. Since the OH group is directly involved in the formation of H-bonds with both neighboring monolinolein molecules and with water molecules in the hydration shell, Sn_3_ and Sn_2_ signals give information about the monolinolein polar head state in *Pn*3̄*m* and *H*_II_ phases, with and without excess water. In addition, the CO–O–C esteric part involves the oxygen lone pairs of the alkoxy group accepting intra- and inter-molecular H-bonds from the OH of the monolinolein and water molecules. Therefore, we can investigate this interaction from the evolution of the CO–O–C stretching signal at 1180 cm^−1^ (Sn_1_).^22,30^

The intense signal from the ester carbonyl located at *ca.* 1730 cm^−1^ is generated by the C=O stretching and is composed of two sub-components. The C=O band that falls at lower frequency (at 1725 cm^−1^) corresponds to carbonyl groups that are more involved in the formation of hydrogen bonds *i.e.*, using both oxygen lone pairs. While the C=O band that falls at a higher frequency (at 1736 cm^−1^) is generated by carbonyl groups that are less involved in the formation of hydrogen bonds *i.e.*, use only one lone pair or none.

**Fig. 5:**
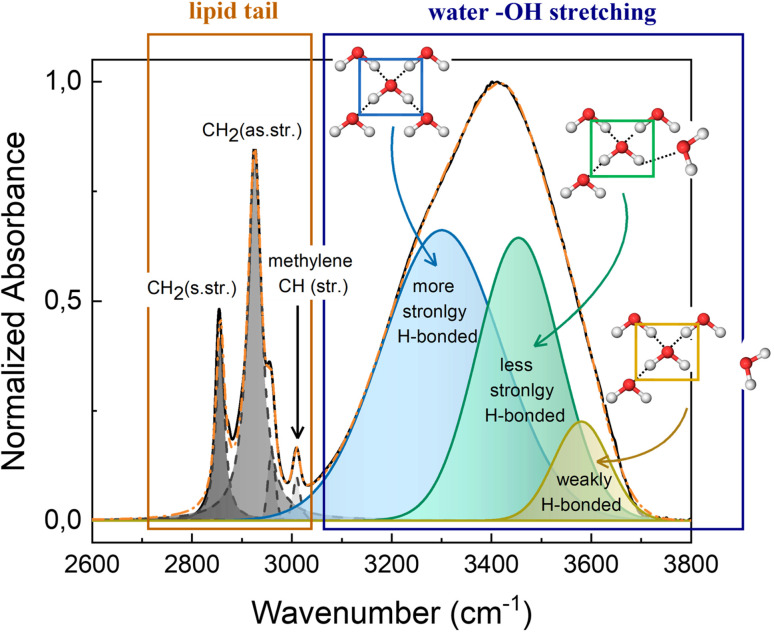
Fitting example of FTIR spectra in the region 2600–3800 cm^−1^ of M70-W30 at 30 °C. The inserted schematics represent the three different types of OH configuration in the water network.

The high wavenumber region (2700–3800 cm^−1^) of the FTIR spectrum contains information on the alkyl chain conformational order and flexibility, as well as on the H-bond network of water. Fig. 5 shows the CH_2_ symmetric and antisymmetric stretching motions that fall at 2856 cm^−1^ and 2924 cm^−1^, respectively, and at about 3010 cm^−1^ the methylene CH stretching is recognizable. The OH stretching signal from 3100 cm^−1^ to 3700 cm^−1^ is a broad band, that results from the contribution of different components. At 3300 cm^−1^ falls the component attributed to the more strongly H-bonded water molecules, *i.e.*, water molecules that are tetrahedrally coordinated with nearby water molecules.^31–33^ Meanwhile, water molecules that are coordinated with other water molecules in a distorted geometry, generate the component at *ca.* 3450 cm^−1^, which represents the less strongly H-bonded water molecules. While at higher wavenumbers (*ca.* 3600 cm^−1^) falls the component attributed to water molecules that are weakly stabilized by H-bond interactions. These three components represent the dynamic equilibrium of the hydrogen bond.^33^ The water network is strongly influenced by changes of thermodynamic parameters and by the presence of other molecules in solution. Thus, the redistribution of the three water fractions gives information about the system response to external stimuli, like pressure and temperature changes, and about the interaction that exists among water molecules and solutes.

### Interaction between monolinolein and water during phase transition

**Fig. 6:**
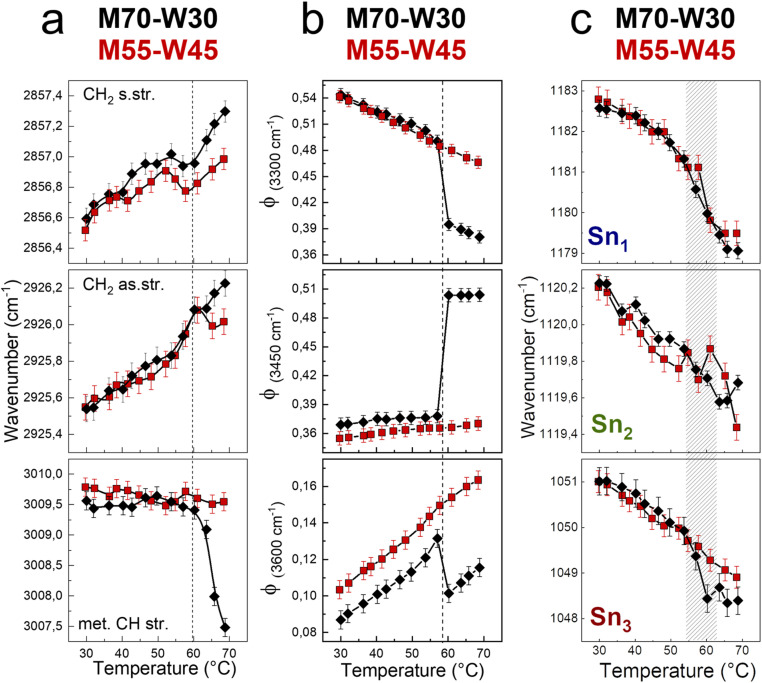
(a) Wavenumber trends of the CH_2_ symmetric, CH_2_ asymmetric, and methylene CH stretching bands. (b) Fraction of strongly H-bonded, less strongly H-bonded, and weak H-bonded water molecules. (c) Wavenumber trends of Sn_1_, Sn_2_, and Sn_3_ bands, as a function of temperature of M70-W30 (black points) and M55-W45 (red points).

We first investigate the effect of excess water on the lipidic mesophase by comparing the alkyl chain conformational order and flexibility, water network, and headgroups during the phase transition between samples with and without excess water, as shown in Fig. 6.

The alkyl chain conformational order and flexibility are investigated using the shift of symmetric and antisymmetric CH_2_ stretching. In both samples, the two signals shift of about 1 cm^−1^ towards high energy as the temperature increases (Fig. 6a). The increases of the vibrational frequencies are the result of the enhancement of the tail motility. Indeed, increasing the temperature, the rotation around the C–C axes is favored, resulting in a transition from *trans* to *gauche* conformers.^34,35^ Compared to those in the *Pn*3̄*m* cubic phase, the lipid tails in the *H*_II_ phase are less densely packed and more mobile, resulting in a higher vibrational energy. However, the methylene CH stretching shows a marked decrease of 2 cm^−1^ of the vibrational frequency in the M70-W30 sample, as a sensitive indicator of the phase transition. As a general trend, the wavenumber shifts that relate to the lipid tails are less pronounced in the presence of excess water (M55-W45). This may be due to the steric hindrance effect. Indeed, excess water interacts with the polar headgroups and acts as a plasticizer. But at the same time, the excess water molecules also occupy a certain space and result in the lipid tails being squeezed compared to the system without excess water.

To quantify changes in the water network, the fractions of different water components are estimated using a fitting procedure and the ratio among the area of each component over the total area of the OH stretching band (*Φ*_*x*_ = *A*_*x*_/*A*_tot_) is shown in Fig. 6b.^36–39^ The three components of OH-stretching (*Φ*_(3300cm^−1^)_, *Φ*_(3450cm^−1^)_ and *Φ*_(3600cm^−1^)_) in bulk water change linearly with temperature: *Φ*_(3300cm^−1^)_ decreases, while *Φ*_(3450cm^−1^)_ and *Φ*_(3600cm^−1^)_ increase. Without excess water, in the M70-W30 sample, all three components show a marked discontinuity at around 55 °C upon heating, which is the temperature of phase transition from the cubic phase to *H*_II_ phase. On the other hand, in the presence of excess water, in the M55-W45 sample, all three OH stretching components show a linear-like trend, because the confined water signal is covered by that of bulk-like excess water.

In general, increasing the temperature results in the increase of lipid flexibility due to the quantity of *trans*–*gauche* rotamers of hydrocarbon chains. The high number of rotamers create the increase of both chains splaying and the spontaneous curvature of the monolayer at the interface.^40^ At the same time, the lateral swell of the lipidic bilayer generates a decrease of both the water channel diameter and the lattice parameter. Therefore, it results in the compression of the lipid headgroups. The compression and denser packing of polar heads at higher temperature is detected from the red shift of Sn_1_, Sn_2_ and Sn_3_ vibrational signals (Fig. 6c). A remarkable change of the wavenumber trend occurs between 55 °C and 62 °C, where both samples experience the transition from the *Pn*3̄*m* cubic phase to *H*_II_ phase.

**Fig. 7:**
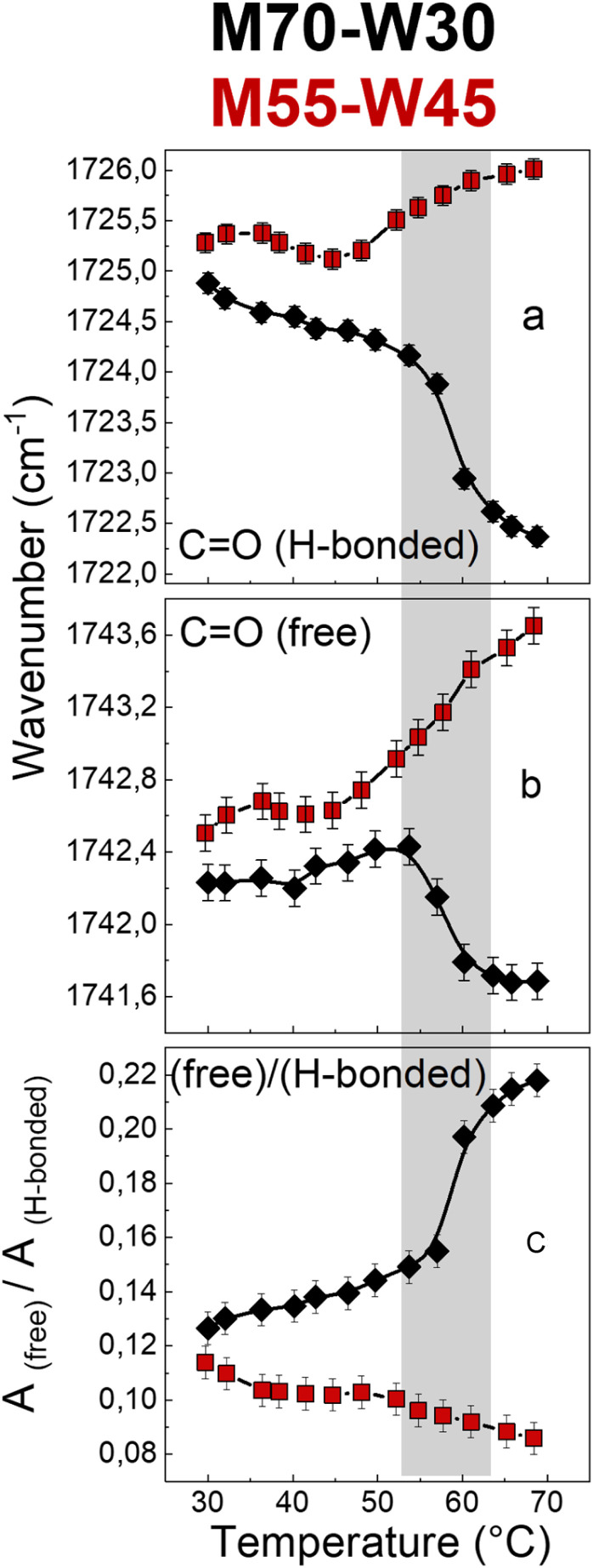
Wavenumber trends of (a) hydrogen-bonded and (b) free C=O bands, and (c) intensity ratio of high (free C=O) to low (H-bonded C=O) frequency components of the carbonyl stretching band of M70-W30 (black points) and M55-W45 (red points).

The carbonyl group plays an important role in understanding the lipid conformation and its interaction with water due to its location at the interface between the polar head and the hydrophobic tail, which is influenced by both the flexibility of the hydrocarbon chain and the rearrangement of the H-bond network between water molecules with –OH and CO–O–C groups of the lipid headgroups. To assess the carbonyl group behaviour during phase transitions and the effect of excess water on it, the carbonyl stretching band has been reproduced using two sub-components which represent two fractions of the C=O oscillators: one more strongly involved in H-bonds and the other less bonded. The fitting results, *i.e.*, sub-component wavenumbers and relative areas, are shown in Fig. 7. Without excess water, in M70-W30 (black points), the wavenumber of the two sub-components decreases with increasing temperature. This is related to a compression effect due to the decrease of the water channel diameter at high temperatures. The polar headgroup compression hinders the stretching of the carbonyl and results in its vibration at lower frequencies. In addition, the phase transition from the *Pn*3̄*m* cubic phase to the *H*_II_ phase leads to denser packing of headgroups, which further compresses the polar heads and disfavours the interaction of the carbonyl with both lone pairs. Therefore, the number of free carbonyls increases while the number of H-bonded carbonyls decreases (Fig. 7a and b).

Interestingly, in the presence of excess water, in M55-W45 (red points), the behaviour of carbonyl is completely the opposite compared to the sample without excess water. The excess water penetrates the interface and forms new H-bonds with other headgroups, therefore, the headgroup packing is much less dense compared to M70-W30. As a result, the two carbonyl sub-components shift to higher wavenumbers with higher vibration energy. This interpretation is further confirmed by the decrease of the free : H-bonded ratio (Fig. 7c). Therefore, without excess water, the headgroups form dense packing during the phase transition. However, if there is the presence of excess water, it can penetrate the interface between the headgroup and tail and accelerate the headgroup mobility as a plasticizer.

## Conclusion

The effect of excess water on dynamics of lipids and their interaction with water were studied in lipidic mesophase during the phase transition from *Pn*3̄*m* cubic phase to *H*_II_ phase. The phase transition temperatures (49–62 °C) of both lipidic mesophases determined from the structural change in SAXS were also observed from BDS and FTIR. The result taken together indicate that not only the headgroups and the tails, but also the interfaces were influenced by the structural change during the phase transition. Both BDS and FTIR confirmed that headgroups were much denser packed in *H*_II_ phase than in *Pn*3̄*m* cubic phase. All the three relaxation processes originating from lipids became faster in the mesophase with excess water, owing to the plasticizing effect of water. In the presence of excess water, the mesophase matrix became faster, thus, the association of headgroups under the matrix became slightly more flexible regardless their denser packing. In addition, the Sn_1_, Sn_2_, and Sn_3_ bands on headgroups had red shift during the phase transition, while the signal from tails had blue shift. This confirmed the denser packing of headgroups and less dense packing of tails in *H*_II_ phase compared to the *Pn*3̄*m* cubic phase. In this work, we discovered that the interface carbonyl group on the lipid is the key to point out the differences of the molecular behaviour between samples with and without excess water during lipidic phase transition. The completely opposite behaviour of carbonyl in the presence of excess water compared to those in mesophases without excess water indicated that the interface between heads and tails on lipids was penetrated by water molecules, forming additional H-bonds. The accumulated water molecules at the interface results in a less densely packing of headgroups in M55-W45 sample and a faster dynamics of the lipidic mesophase.

## Experimental section

### Materials and sample preparation

Dimodan U/J (commercial grade monolinolein) containing >98 wt% monoglyceride was a generous gift from Danisco, Denmark, and was used as received. The Milli-Q water was taken from a Millipore purification system operating at a resistivity of 18.2 MΩ cm. The lipidic mesophase samples were prepared by mixing monolinolein with water using a home-built setup with two connected Hamilton RN syringes. Before mixing, one syringe was loaded with the desired amount of monolinolein, and the other was loaded with the corresponding amount of water. The homogeneous mesophase was prepared after mixing 100 times through the connector.

### Small angle X-ray scattering (SAXS)

SAXS profiles of monolinolein–water mesophases were acquired with a Bruker AXS Micro X-ray operated at a voltage of 50 kV and a current of 1000 μA. Measurements were performed with Cu Kα radiation (*λ* = 1.5418 Å) collimated by a 2D Kratky collimator. The SAXS profiles were collected with a 2D Pilatus K100 detector. The lipidic mesophase sample was sealed in capillaries 1.5 mm in diameter. Measurements were carried out every 4 °C from 30 to 66 °C and samples were equilibrated for 30 min before exposure for 15 min. The scattering vector was calibrated with silver behenate.

### Broadband dielectric spectroscopy (BDS)

The BDS measurements were performed on a Novocontrol Alpha frequency analyzer covering the frequency range 10^−2^ to 10^6^ Hz. Measurements were carried out with parallel plate geometry, with a top electrode (diameter: 20 mm) and bottom electrode (diameter: 40 mm). The sample thickness was maintained using a teflon spacer with thickness 50 μm. The frequency dependence spectra were obtained on heating from 31 to 70 °C with a step of 3 °C. The sample was equilibrated for 30 min at each temperature before the spectrum was taken. The obtained complex dielectric permittivity (ε*), ε* = *ε*′−i*ε*′′ (*ε*′ is the real part, *ε*′′ is the imaginary part), is a function of frequency (*f*), temperature (*T*), and pressure (*P*), ε* = ε* (*f*, *T*, *P*). We used a summation of several Havriliak and Negami (HN) equations to analyse the spectra. The HN equation is described as follows:1
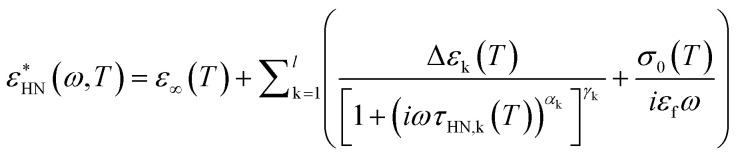
where k indicates the individual relaxation process, Δ*ε*_k_(*T*) is the relaxation strength at temperature *T*, *τ*_HN,k_ is the characteristic relaxation time, *α*_k_, *γ*_k_ (0 < *α*, *αγ* ≤ 1) describe the symmetrical and asymmetrical broadening of the process and *ε*_∞_ is the dielectric permittivity at the limit of high frequencies. At lower frequencies, *ε*′′ rises due to the conductivity (*ε*′′ = *σ*/(*ωε*_f_), where *σ* is the dc conductivity and *ε*_f_ the permittivity of free space). The conductivity contribution was also considered during the fitting process.

The relaxation times at maximum loss (*τ*_max_) were obtained from *τ*_HN_ following:2
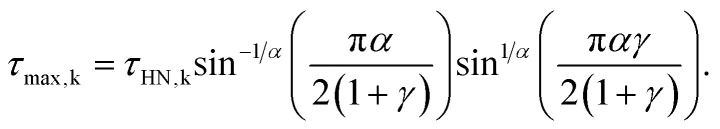


The loss tangent functions (tan *δ*) were fitted combining eqn (1) and (2). However, the frequencies of the maximum peak positions are subtly shifted compared to the representation of the dielectric loss (*ε*′′). In the case of the single Debye process with no dc conductivity:3
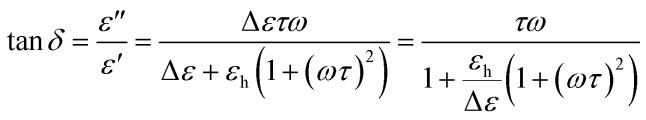
where *ε*_h_ is the high frequency limit of the dielectric permittivity. Eqn (3) leads to a peak maximum when *ωτ* = (1 + Δ*ε*/*ε*_h_)^1/2^.

### Fourier transform infrared spectroscopy (FTIR)

The FTIR spectra were acquired using a Varian 640 FTIR spectrometer in transmission configuration under a N_2_ atmosphere. The average of 64 scans were acquired from 1000 cm^−1^ to 4000 cm^−1^ with a resolution of 2 cm^−1^. The background was subtracted automatically. Samples were sandwiched and sealed between two CaF_2_ windows and the 6 μm sample thickness retained using a spacer. Measurements were carried out every 3 °C from 28 to 70 °C. The temperature was controlled by a Julabo thermal bath. For accurate control of sample temperature, an external thermocouple was placed next to the CaF_2_ windows where the sample was sandwiched. The samples were equilibrated for 30 min before measurement at each temperature.

## Author contributions

Y. Y. and S. C. conceived the idea and planned the experiments. Y. Y. performed the SAXS and BDS measurements, analyzed and interpreted the data. S. C. performed the FTIR measurements and analyzed and interpreted the data. All the authors discussed the results, wrote, and revised the manuscript.

## Conflicts of interest

The authors declare no conflict of interest.

## Supplementary Material

FD-249-D3FD00118K-s001
