# Supplementary material for: Water–lipid interface in lipidic mesophases with excess water
Source: Faraday Discuss. 2023 Jun 29;249:469–84. doi: 10.1039/d3fd00118k (PMC10845009; doi:10.1039/d3fd00118k)
Supplement: FD-249-D3FD00118K-s001 [file FD-249-D3FD00118K-s001.pdf]

Electronic Supplementary Material (ESI) for Faraday Discussions.
This journal is © The Royal Society of Chemistry 2023

Supporting Information

Water-Lipid Interface in Lipidic Mesophases with Excess Water

Yang Yao,[1a] Sara Catalini,[2,3a] Paolo Foggi,[2,3] and Raffaele Mezzenga[1,4*]

[1]*Department of Health Sciences and Technology, ETH Zürich, 8092 Zurich, Switzerland*

[2]*European Laboratory for Non-Linear Spectroscopy, LENS, 50019, Firenze, Italy*

[3]*Department of Chemistry, University of Perugia, Via Elce di Sotto 8, 06123 Perugia, Italy*

[4]*Department of Materials, ETH Zurich, 8093 Zürich, Switzerland*

[a] These authors contributed equally: Yang Yao, Sara Catalini

[*]Authors for correspondence: E-mail: raffaele.mezzenga@hest.ethz.ch

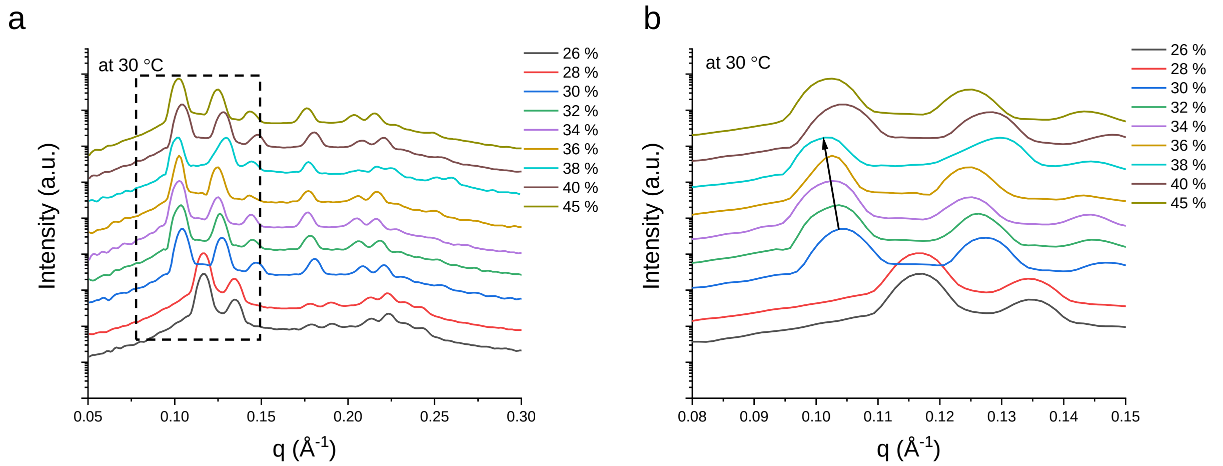

Figure S1. (a) Comparison of SAXS profiles of monolinolein-based lipidic mesophase with different water fractions obtained at 30 °C. Figure S2 (b) is the magnification of (a) in the dashed frame region.

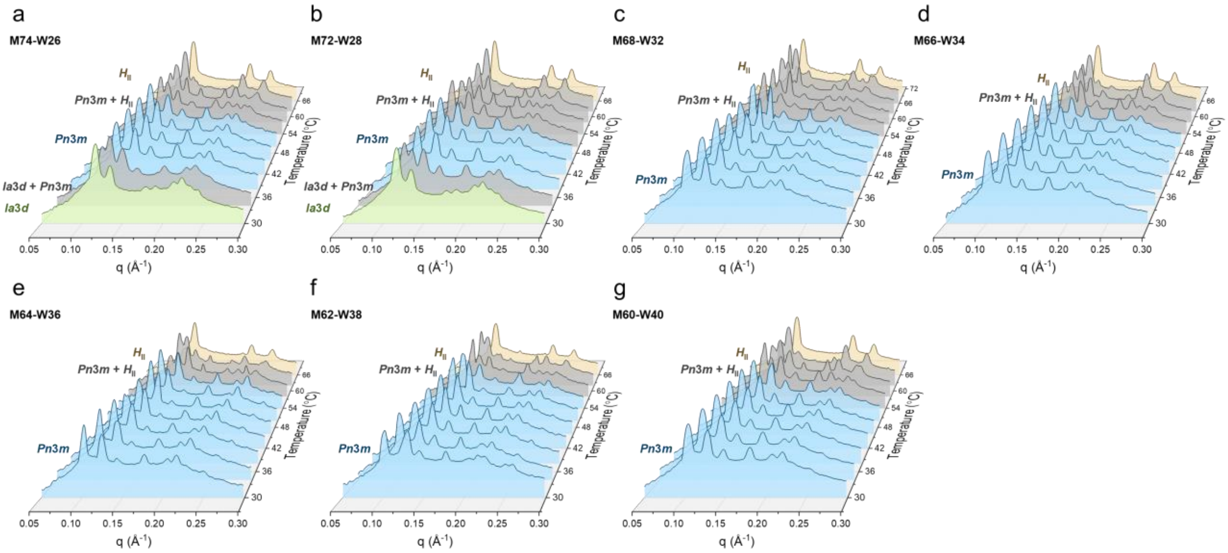

Figure S2. SAXS profiles of (a) M74-W26, (b) M72-W28, (c) M68-W32, (d) M66-W34, (e) M64-W36, (f) M62-W38, and (g) M60-W40 as a function of temperature.

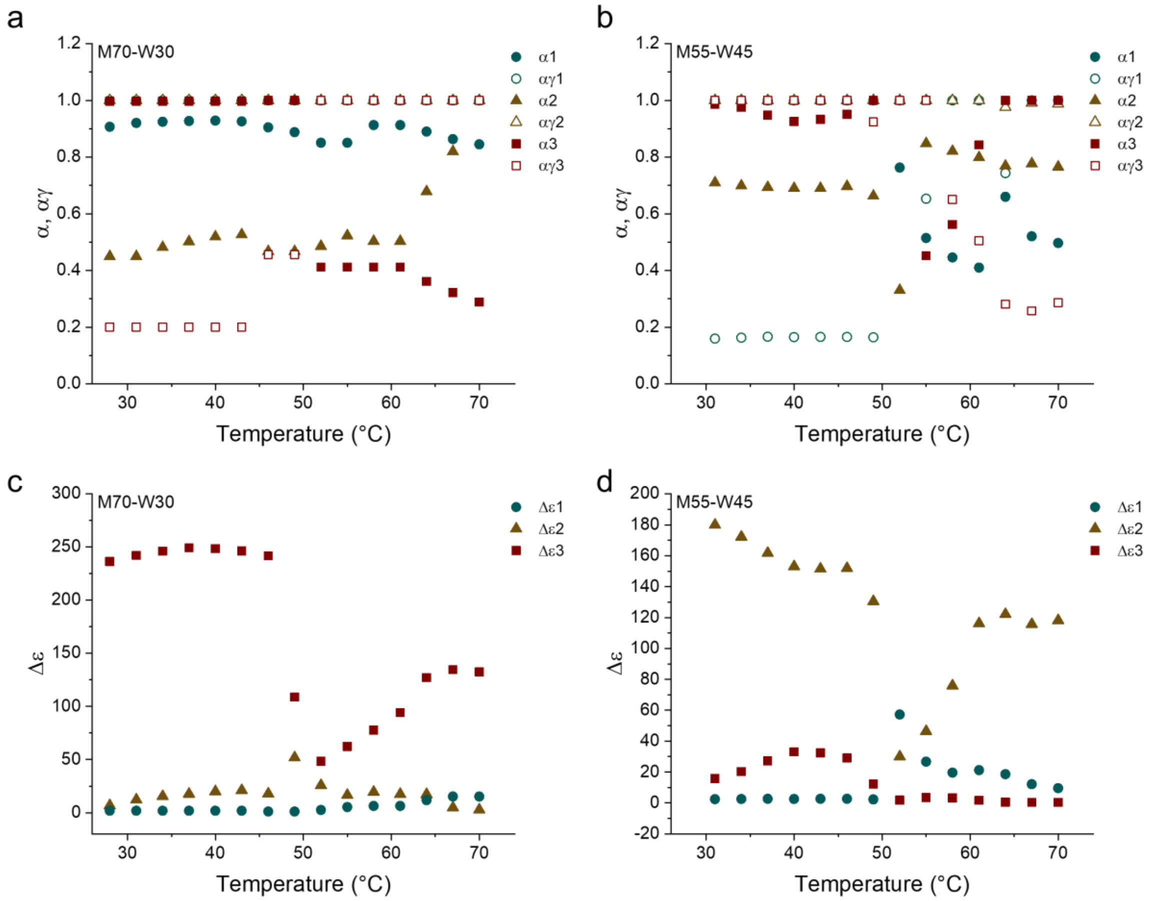

Figure S3. α, αγ parameters obtained from fitting of dielectric spectra for (a) M70-W30 and (b) M55-W45. Intensity of dielectric loss (Δε) obtained from fitting of dielectric spectra for (c) M70-W30 and (d) M55-W45.
